# Dual-radionuclide in vivo imaging of micro-metastasis and lymph tract with submillimetre resolution

**DOI:** 10.1038/s41598-023-46907-1

**Published:** 2023-11-09

**Authors:** Atsushi Yagishita, Shin’ichiro Takeda, Kazunobu Ohnuki, Miho Katsuragawa, Oltea Sampetrean, Hirofumi Fujii, Tadayuki Takahashi

**Affiliations:** 1 Kavli Institute for the Physics and Mathematics of the Universe (Kavli IPMU, WPI), The University of Tokyo, 5-1-5 Kashiwanoha, Kashiwa, Chiba 277-8583 Japan; 2 Exploratory Oncology Research and Clinical Trial Center, National Cancer Center, 6-5-1 Kashiwanoha, Kashiwa, 277-8577 Japan; 3 Department of Molecular Biology, Keio University School of Medicine, 35 Shinanomachi, Shinjuku, Tokyo, 160-8582 Japan; 4 Human Biology-Microbiome-Quantum Research Center (WPI-Bio2Q), Keio University, 2-15-45 Mita, Minato, Tokyo, 108-8345 Japan; 5 Department of Physics, The University of Tokyo, 7-3-1 Hongo, Bunkyo, Tokyo, 113-0033 Japan

**Keywords:** Imaging, Molecular imaging

## Abstract

Multi-radionuclide in vivo imaging with submillimetre resolution can be a potent tool for biomedical research. While high-resolution radionuclide imaging faces challenges in sensitivity, multi-radionuclide imaging encounters difficulty due to radiation contamination, stemming from crosstalk between radionuclides and Compton scattering. Addressing these challenges simultaneously is imperative for multi-radionuclide high-resolution imaging. To tackle this, we developed a high-spatial-resolution and high-energy-resolution small animal single-photon emission computed tomography (SPECT) scanner, named CdTe-DSD SPECT-I. We first assessed the feasibility of multi-tracer SPECT imaging of submillimetre targets. Using the CdTe-DSD SPECT-I, we performed SPECT imaging of submillimetre zeolite spheres absorbed with ^125^I^-^ and subsequently imaged ^125^I-accumulated spheroids of 200–400 µm in size within an hour, achieving clear and quantitative images. Furthermore, dual-radionuclide phantom imaging revealed a distinct image of the submillimetre sphere absorbed with ^125^I^-^ immersed in a ^99m^Tc-pertechnetate solution, and provided a fair quantification of each radionuclide. Lastly, in vivo imaging was conducted on a cancer-bearing mouse with lymph node micro-metastasis using dual-tracers. The results displayed dual-tracer images of lymph tract by ^99m^Tc-phytic acid and the submillimetre metastatic lesion by ^125^I^-^, shown to align with the immunofluorescence image.

## Introduction

Molecular imaging using multiple fluorescent probes that target specific molecules is indispensable in cell biology. Similarly, high-resolution molecular imaging using radioactive tracers targeting multiple molecules is a potent tool for biomedical research. Molecular imaging is used to detect and analyse target molecules and multi-tracer (probe) imaging is used to elucidate the relationships between them. Although multi-tracer imaging with fluorescent dyes of various colours is a valuable approach for *in vitro* experiments, it faces challenges for *in vivo* imaging of deep animal tissues because of the absorption of most visible light. In contrast, because radiation can penetrate these tissues, radioactive tracers are more suitable for *in vivo* imaging of deep tissues in animals.

There are two main modalities for molecular imaging using radioactive tracers: single-photon emission computed tomography (SPECT) and positron emission tomography (PET). Notably, SPECT is suitable for multi-tracer imaging. Some clinical reports have described the use of dual SPECT tracers with millimetre-level spatial^1–5^. PET is also suitable for multi-tracer imaging using prompt-gammas^6^. However, SPECT can achieve a higher spatial resolution of less than 500 µm^7,8^. Achieving a few hundred micrometres in spatial resolution could elucidate molecular localization, molecular function or physiological activity in relation to fine structure at a few hundred micrometres level. However, combining multi-tracer and high-resolution SPECT presents substantial technical challenges.

A higher spatial resolution leads to smaller voxel volumes, which in turn capture fewer photons per voxel, thereby extending the acquisition time. Given the potential adverse effects of anaesthesia on animals, shorter acquisition times are preferable for *in vivo* imaging. Another challenge is spectral contamination due to factors such as Compton scattering and fluorescent X-rays of the collimators and other tracers in multi-tracer setups.

To address these problems, we developed a SPECT system equipped with cadmium telluride (CdTe) detectors and a multi-pinhole collimator, named CdTe-DSD SPECT-I^8^ for small animal imaging. This system boasts both high spatial resolution (350 µm or better) and high energy resolution. Notably, low-energy X-rays are beneficial to create high-spatial-resolution pinhole collimators, unlike their higher-energy counterparts that can penetrate collimators more readily. In SPECT imaging of small animals for life science studies and drug development (other than clinical diagnostic radiopharmaceuticals), ^125^I, which emits low-energy (27.5 keV) X-rays, is frequently chosen as the preferred radioactive iodine source. This is largely owing to its extended half-life (approximately 60 days) compared to ^123^I, which has a half-life of 13.2 h, rendering ^125^I more practical for laboratory studies. Given the diminutive size of mice and other such small animals, the effects of attenuation are not as pronounced as in larger animals. A distinct feature of ^125^I is its use in long-term follow-up tracer experiments. Conventional SPECT has a low-energy resolution for low-energy X-rays (i.e. a low signal-to-noise ratio) and is not suitable for multi-radionuclide imaging with ^125^I. Harnessing low-energy X-rays and utilising CdTe-DSD SPECT-I, which has high-spatial resolution and high-energy resolution, enables the visualisation of the microstructure of small animal tissues by multi-tracer *in vivo* imaging, which has been difficult to achieve with conventional SPECT and fluorescence imaging.

In our previous study, we demonstrated that the CdTe-DSD SPECT-I system is capable of high-resolution multi-radionuclide imaging in both *in vitro* and *in vivo* trials involving normal mouse tissues of several millimetres. These tests were optimised by adjusting the radionuclide doses to evaluate the spatial and energy resolutions. However, for *in vivo* imaging of submillimetre tissues, the radiation dose in the target tissue is expected to be quite low owing to its size. Thus, achieving sufficient photon counts within a practical acquisition period remains challenging. This complexity is further compounded in multi-radionuclide imaging owing to potential noise signals from other radiation sources.

To assess the feasibility of multi-radionuclide imaging for microscopic targets, we focused on the practical application of sentinel lymph node biopsy. This procedure is crucial for early stage breast cancer diagnosis, in which sentinel lymph nodes are excised and pathologically examined for lymph node^9,10^. The sentinel lymph node is the primary site where cancer cells are likely to spread from a primary tumor^11^. Although SPECT can identify sentinel lymph nodes, it is currently difficult to simultaneously identify metastatic lesions, particularly micro-metastases.

We, therefore, aimed to explore the feasibility of *in vivo* dual-radionuclide imaging of submillimetre tissues. We adopted a step-by-step approach, starting with *in vitro* trials involving phantoms and spheroids, and moved to dual-tracer images of lymph node micro-metastasis and lymph tracts in a cancer-bearing mouse, which images the early stages of cancer metastasis simultaneously with the metastatic pathway. Thus, in contrast to conventional SPECT and fluorescence imaging, our method can visualise the microstructure of small-animal tissues by *in vivo* imaging using multi-tracer imaging.

## Results

### Sub-millimetre phantom imaging

**Figure 1:**
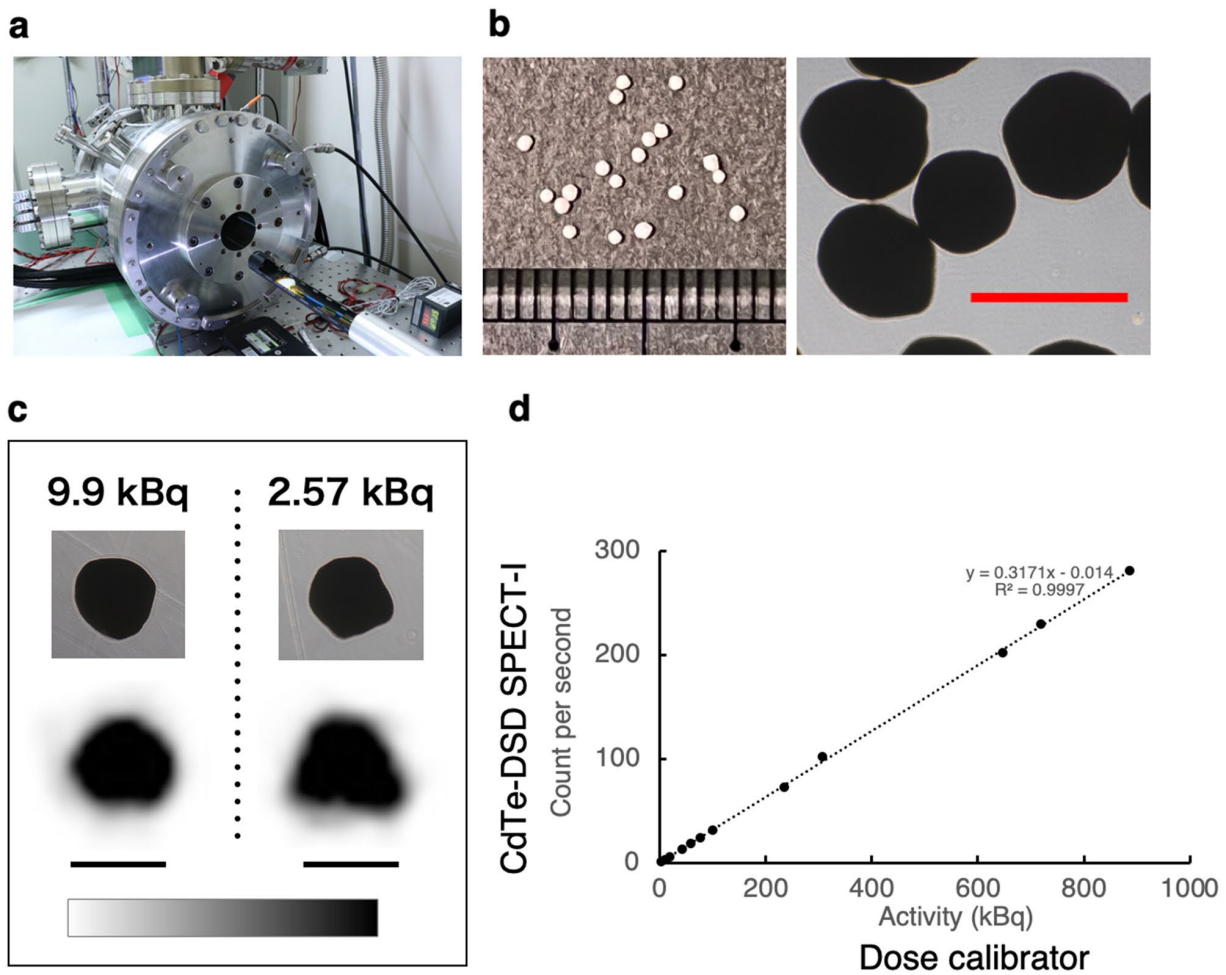
(**a**) SPECT system employed in this study. (**b**) Silver zeolite spheres. The right figure shows a microscopic image. Scale bar: 1.0 mm. (**c**) Microscopic images (Top) and representative SPECT images (Bottom) of the zeolite spheres with absorbed ^125^I^-^. Both microscopic and SPECT images are at the same scale. Scale bar: 1.0 mm. (**d**) Plot illustrating the strong linear correlation between the measured activity using a dose calibrator and count per second obtained with CdTe-DSD SPECT-I.

We evaluated the imaging performance of submillimetre non-living samples with varied radioactivity levels. Achieving sufficient photon counts to produce a clear image within an hour is essential, particularly when the activity is as low as a few kBqs. This is because prolonged anaesthesia for small animals in in vivo imaging is harmful and, ideally, acquisition times should not exceed an hour. The SPECT system used, the CdTe-DSD SPECT-I, is shown in Fig. 1a. We adsorbed radioactive iodine (^125^I) in the form of a ^125^I-NaI aqueous solution, with radiation doses varying from 2.57 kBq to 886 kBq, onto silver zeolite spheres smaller than 1.0 mm (Fig. 1b). Imaging of these 12 samples took 10 min each. Figure 1c shows SPECT images of the samples with activities of 9.9 kBq and 2.57 kBq. A strong correlation was observed between the photon counts from the SPECT system and the activity measured using a dose calibrator, as shown in Fig. 1d (r^2^ = 0.9997). Supplementary Table 1 provides detailed data for all the zeolite spheres. We achieved quantitative imaging with submillimetre samples, even at low kBq levels, within 10 min.

### Spheroid imaging

**Figure 2:**
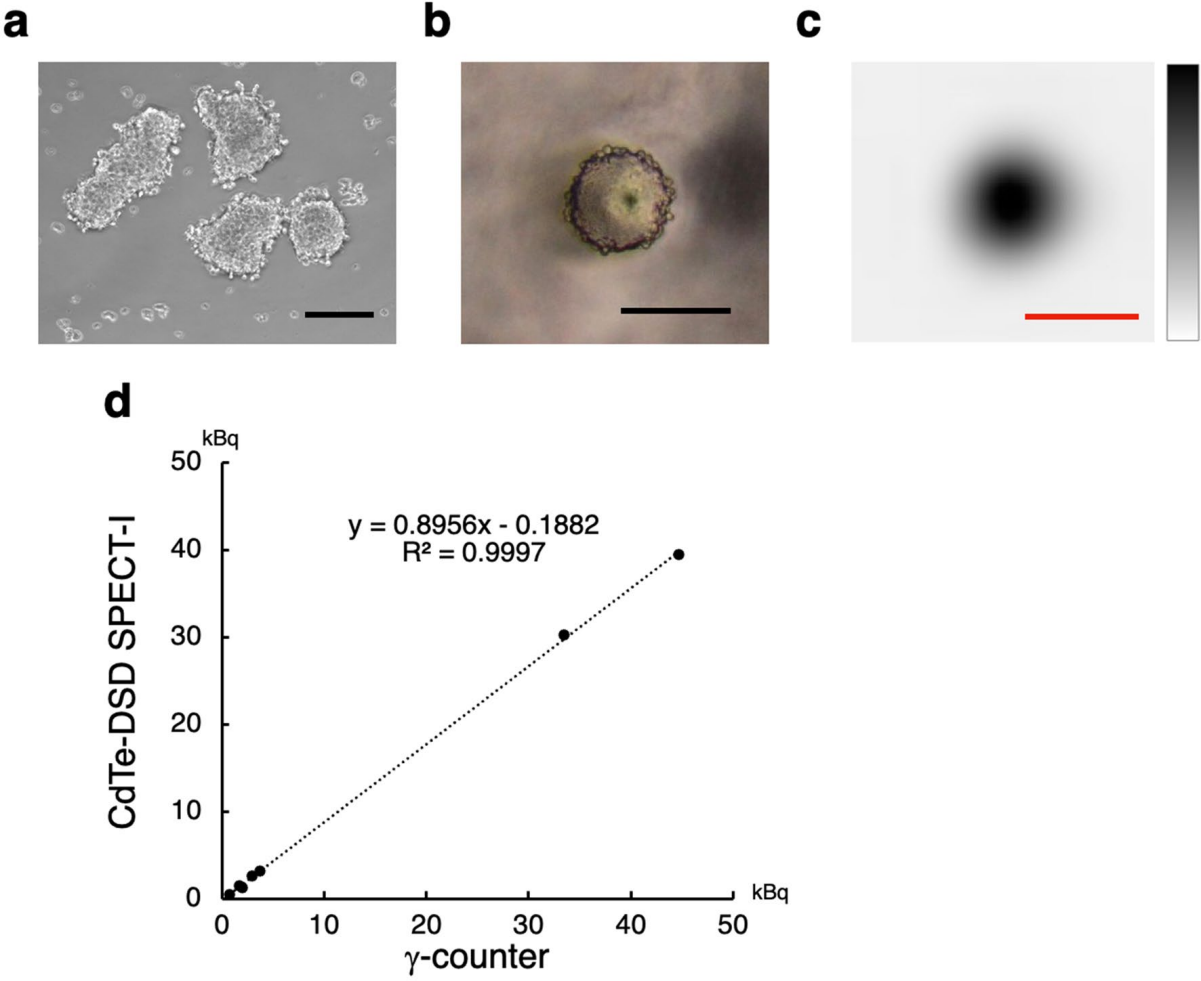
(**a**) 4T1-mNIS cell Spheroids. Scale bar: 200 µm. (**b**) Microscopic view of an isolated spheroid prepared for SPECT imaging. Scale bar: 200 µm. (**c**) SPECT image of the spheroid incorporating 0.77 kBq of ^125^I^-^. Scale bar: 500 µm. (**d**) Plot comparing spheroid activity measured by γ-counter and CdTe-DSD SPECT-1, illustrating a strong linear correlation.

Next, we aimed to image spheroids approximately 200 µm in size, matching the spatial resolution of CdTe-DSD SPECT-I. We cultured 4T1 cells (a mouse mammary tumour cell line) overexpressing a murine sodium/iodide symporter (4T1-mNIS) to form spheroids (Fig. 2a). These 200–400 µm spheroids were incubated with ^125^I-NaI-containing medium and washed three times with Hanks’ balanced salt solution (HBSS). Each spheroid was collected under a microscope using a small amount of HBSS (Fig. 2b). The acquisition time was set depending on the anticipated activity based on factors such as the size of the spheroid, to comfortably exceed a count of 2.4 × 10^4^, ensuring a statistical error of less than 2%, with a maximum duration of 60 min. Figure 2c shows a SPECT image of a spheroid sized 210 × 181 µm with 0.77 kBq activity. There was a strong correlation between activity from the region of interest (ROI) in the SPECT images and γ-counter measurements, as evidenced in Fig. 2d (r^2^ = 0.9997). Supplementary Table 2 contains detailed spheroid data, demonstrating that quantitative SPECT imaging of biological samples as small as 200 µm was feasible within an hour. The spheroids with the lowest activity (0.77 kBq) had 3.84 × 10^5^ counts per 60 min. This suggests that even a five-minute imaging session was sufficient to obtain 2.4 × 10^4^ counts.

### Dual-radionuclide imaging of phantom model

**Figure 3:**
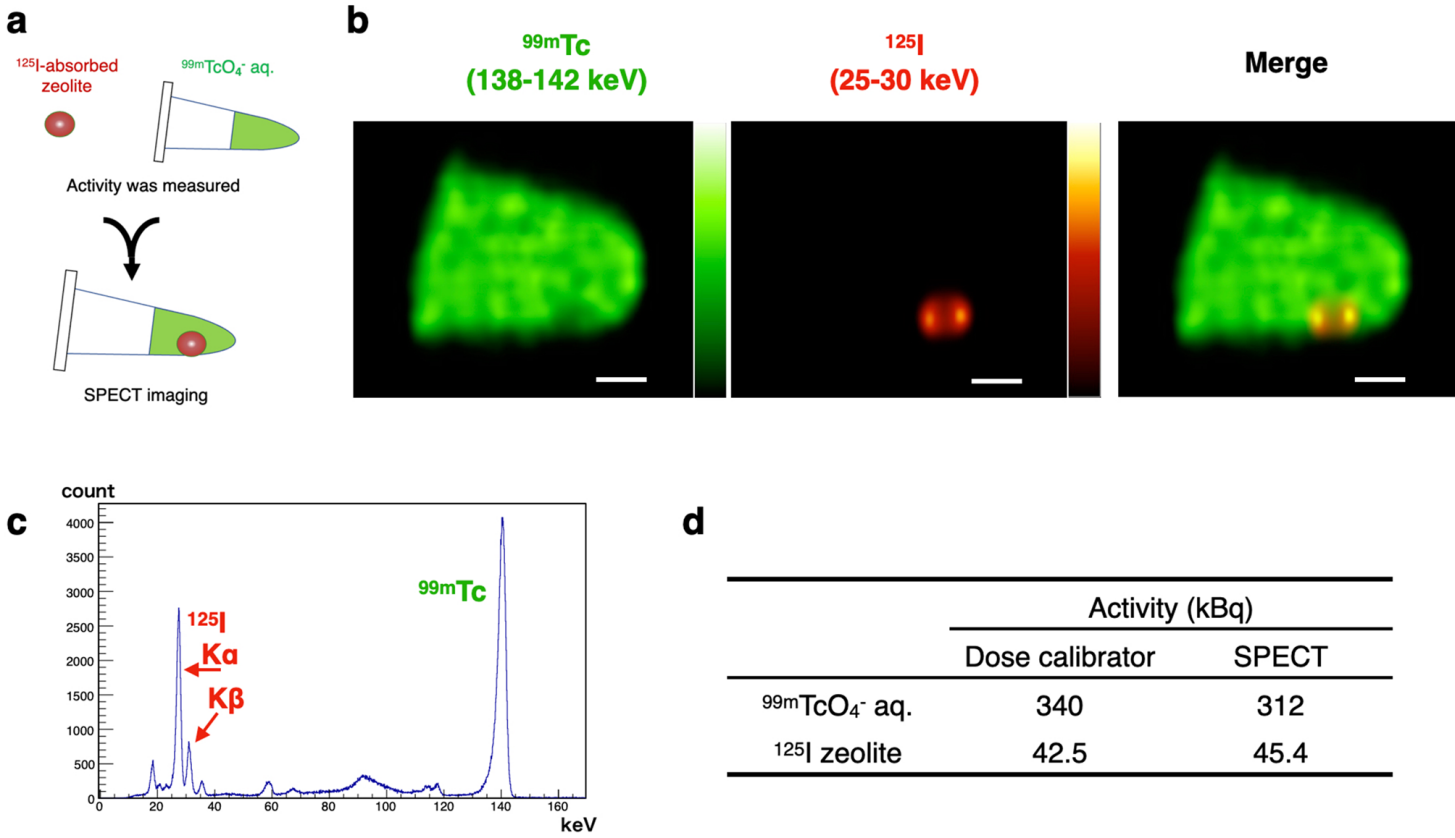
(**a**) Illustrated explanation of the experiment in this section. (**b**) SPECT images of the phantom. Photons in the ^125^I energy band (25–30 keV) are coloured red and photons in the ^99m^Tc energy band (138–142 keV) are coloured green. Scale bar: 1.0 mm. (**c**) Spectrum obtained by phantom imaging. (**d**) Phantom activity comparison: activity obtained from dual-radionuclide SPECT images versus those measured individually with a dose calibrator.

Phantom imaging was performed to assess the potential of dual-radionuclide imaging for micro-metastases and lymph tract visualisation. We used an aqueous solution of ^99m^Tc-pertechnetate as a surrogate lymph tract tracer and silver zeolite with a size of 787 µm absorbed with ^125^I^-^ as a metastatic tumour marker (Fig. 3a). After measuring the activities of both the solution and zeolite using a dose calibrator, they were transferred to a microtube. Figure 3b shows the SPECT images of the phantom obtained after an acquisition time of 60 min. In the ^99m^Tc-pertechnetate solution, a submillimetre object containing ^125^I^-^ was distinctly visible. Figure 3c depicts the spectra of the phantom, where both the Kα (27.5 keV) and Kβ (31.0 keV) emission lines of ^125^I can be distinctly discerned owing to the high-energy resolution of the CdTe semiconductor detector. Finally, Fig. 3d compares the activities derived from the dual-radionuclide images with those measured individually using a dose calibrator. The difference in the activity levels between the two groups was less than 10%.

### Dual-radionuclide in vivo imaging of micro-metastasis and the lymph tract

**Figure 4:**
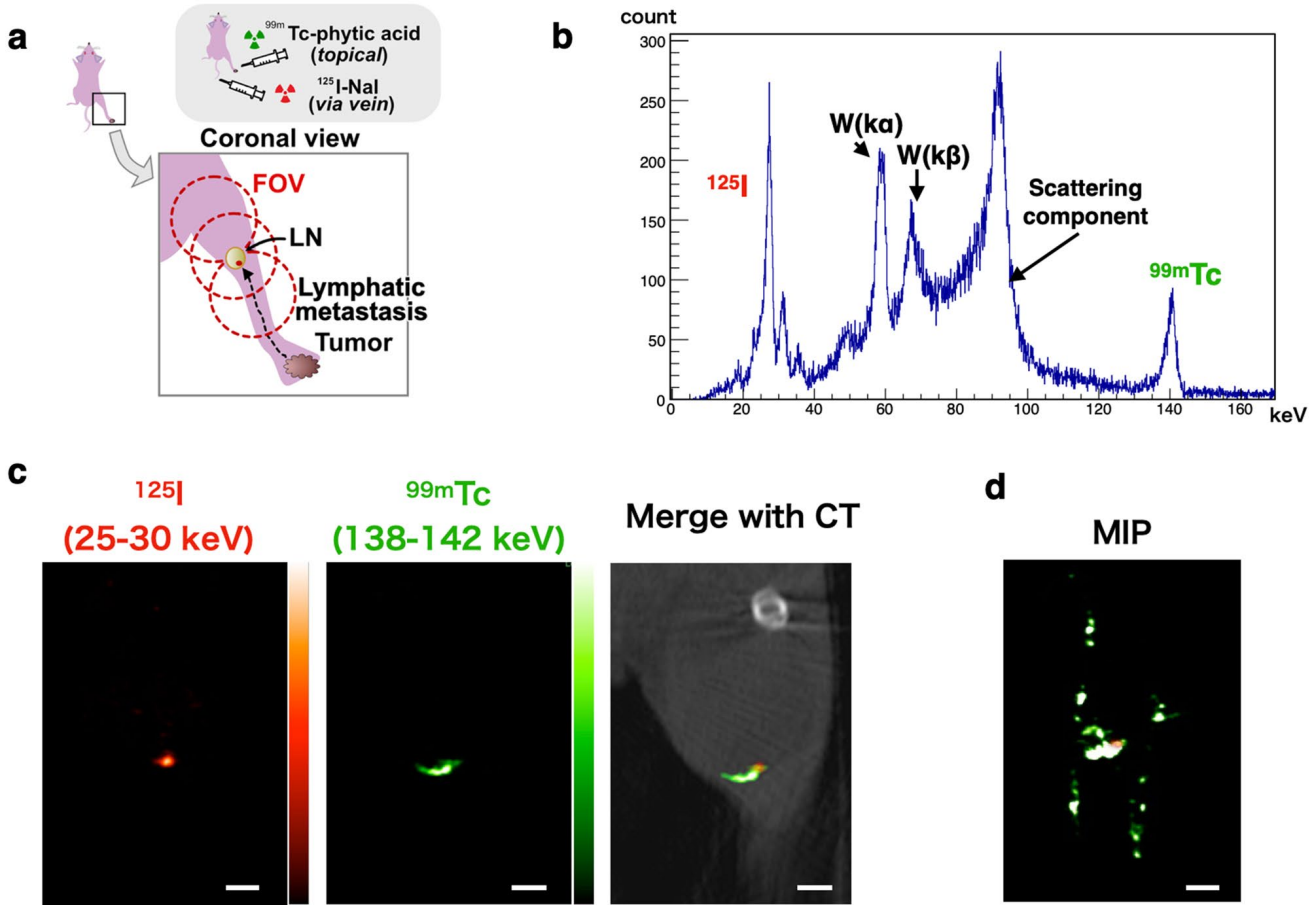
(**a**) Illustrated explanation of the experiment in this section. Image data were obtained with CdTe-DSD SPECT-I, focusing on the popliteal lymph node (LN) where metastasis was anticipated. The red dashed circles indicate the fields of view (FOVs) for SPECT. (**b**) Spectrum obtained with CdTe-DSD SPECT-I. Distinct spectral peak for the Kα line of ^125^I (27.5 keV) and ^99m^Tc (140 keV) are evident. W(Kα/β) indicates fluorescent X-rays of tungsten. (**c**) SPECT(/CT) image slices of the right leg of the cancer-bearing mouse in the coronal view (**a**). Photons in the ^125^I energy band (25–30 keV) are coloured red and those in the ^99m^Tc energy band (138–142 keV) are coloured green. Scale bar: 2.0 mm. (**d**) SPECT maximum intensity projection (MIP) image corresponding to the image in (**c**). The green ductal structure indicates the lymphatic tract, and the adjacent red spot indicates a metastatic tumour. Scale bar: 2.0 mm.

As a result, we conducted dual-radionuclide *in vivo* imaging. Figure 4a illustrates the experimental setup. We transplanted 4T1-mNIS cancer cells into the footpad, and performed SPECT imaging 4 weeks post-inoculation, coinciding with the expected metastasis^12^ to the ipsilateral popliteal lymph node (sentinel lymph node). We administered ^125^I -NaI intravenously as a tumour tracer and ^99m^Tc-phytic acid topically around the tumour as a lymph tracer^13^. The acquisition time per field of view (FOV) was set to 30 min, and the imaging range was centred around the lymph node with 3 FOV coverage. Figure 4b shows the spectrum obtained with CdTe-DSD SPECT-I, showing distinct spectral peaks of ^125^I (27.5 keV) and ^99m^Tc (140 keV), with more scattering components than those observed in phantom imaging (Fig. 3c). Figure 4c shows coronal SPECT/CT images and Fig. 4d shows the SPECT maximum intensity projection (MIP) image. A green ductal structure indicative of ^99m^Tc-phytic acid was observed in the craniocaudal direction, which indicates the lymphatic tract. Adjacent to this ductal structure is a red object less than 1 mm in size representing ^125^I, which indicates a metastatic tumour. The activity and the activity concentration of ^125^I from the image of this object, suggestive of a metastatic tumour, including uncertainties derived from photon statistics and image reconstruction, was 1.10 ± 0.06 kBq and 11.6 ± 0.6 kBq/mm^3^, respectively. This value underestimated the true value by approximately 20% because it did not take attenuation into account (see Supplementary Method). The method used to calculate the uncertainties from the simulations is detailed in the Supplementary Method. Supplementary Fig. 1 verifies the appearance of the image during the lower activity of this tumour by simulation using the data from this animal experiment and zeolite sphere data. At 100 Bq (0.1 kBq), the difference from the surroundings was discernible, whereas at 50 Bq (0.05 kBq), the image was not discernible from the background.

**Figure 5:**
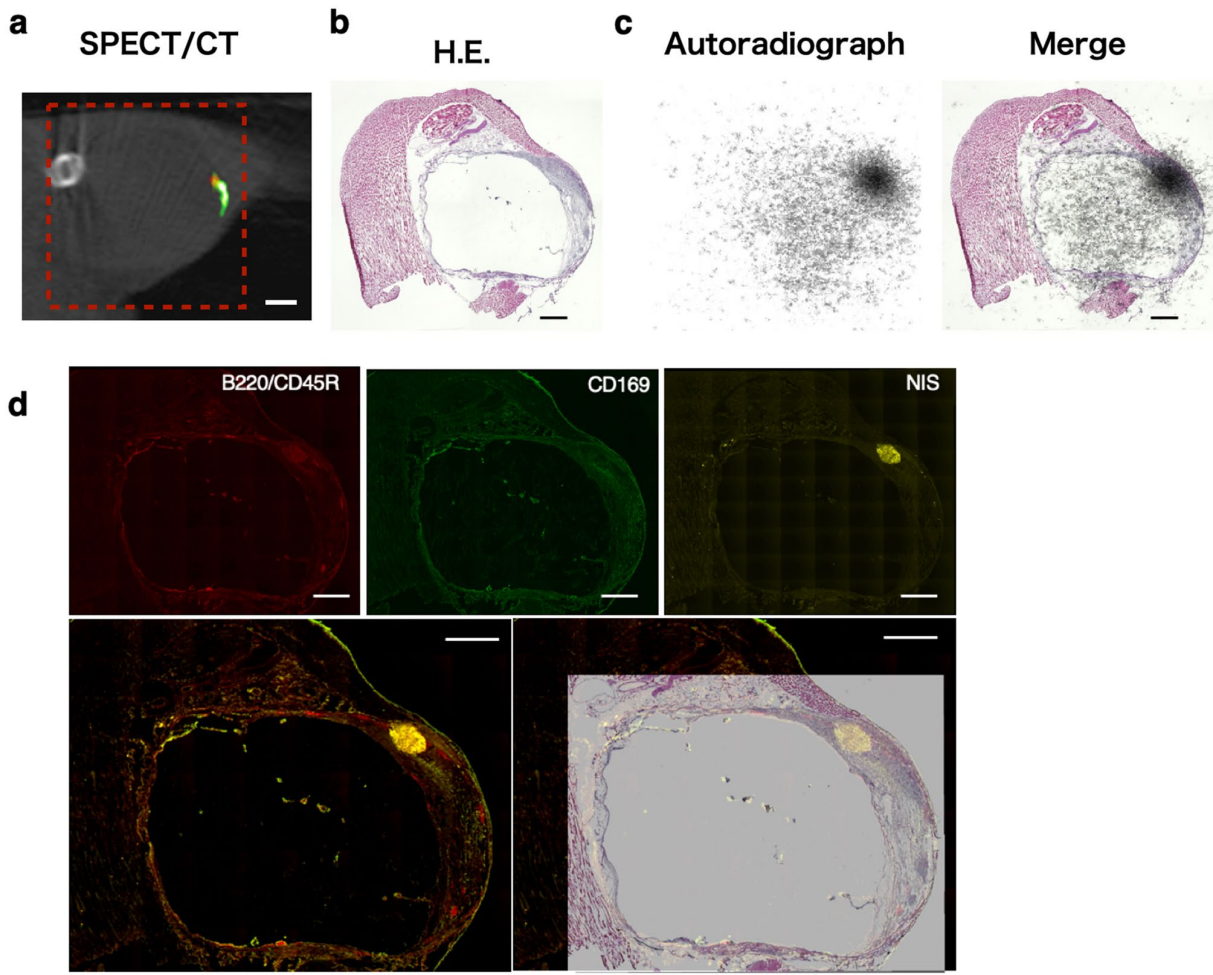
(**a**) SPECT/CT image shown in Fig. 4c rotated 90° anti-clockwise for comparative purposes. The red dashed square indicates the excision line intended for the tissue sample for histological analysis. Scale bar: 2.0 mm. (**b**) Haematoxylin and eosin (H&E) staining of the popliteal lymph node (LN) and the surrounding tissues. The LN is identifiable by the appearance of a dilated vacuole. Scale bar: 1.0 mm. (**c**) An autoradiograph of a serial section, showing a high-intensity spot located at the marginal sinus of the LN. (**d**) Immunofluorescence staining of the serial section. B220/CD45R-positive cells indicate B cells and CD169-positive cells indicate macrophages typically found in the marginal sinus of the LN. The merged images (Bottom images) show that the NIS-positive tumour, less than 1.0 mm in size, is located in the marginal sinus of LN, which is consistent with both the autoradiograph and the SPECT/CT image. Scale bar: 1.0 mm.

To confirm that this tiny red object was a metastatic lymph node tumour, we performed a detailed examination. The lymph node and its surrounding tissue were excised *en bloc* and subjected to histological analysis and comparison with SPECT images. A 90° rotated image of Fig. 4c is shown for reference in Fig. 5a. The haematoxylin and eosin (H&E)-stained image, corresponding to the coronal SPECT view shown in Fig. 5a, is displayed in Fig. 5b; here, the distention of the lymph node is evident. Autoradiography of selected serial sections revealed a high-intensity spot in the marginal sinuses of the lymph node (Fig. 5c). Immunofluorescence images from serial sections (Fig. 5d) clearly depict an NIS-positive tumour smaller than 1 mm, consistent with both the autoradiograph and SPECT/CT imagery. The results for another mouse are presented in Supplementary Fig. 2, although the metastatic tumour was > 1 mm in size.

## Discussion

We investigated the feasibility of dual-radionuclide imaging of micro-metastases and lymph tracts in tumour-bearing mice using two specific tracers. We obtained clear SPECT images of both the micro-metastatic tumour and the lymph tract. Immunohistochemical analysis corroborated that the SPECT images mirrored the histological features, which was consistent with the observations in multi-probe fluorescence molecular imaging. Imaging of a dual-radionuclide mixture sample using a phantom containing microtargets of less than 1.0 mm was considered to validate the SPECT images and quantify them. Finally, dual-radionuclide SPECT images corresponding to weakly magnified multi-probe fluorescence immunostaining images were obtained, although the *in vivo* quantification was not precise because attenuation correction was not performed. As an imaging modality for small animals in the laboratory, the proposed approach can, to some extent, overcome the limitations of high-spatial-resolution multi-tracer *in vivo* imaging using fluorescence imaging and conventional SPECT.

High-energy resolution helps reduce spectral contamination from tracers labelled with different radionuclides to some extent by reducing the spectral overlap (crosstalk) of the emission lines in some cases, especially when the energies of these lines are close. In this study, we employed ^99m^Tc (140 keV) and ^125^I (Kα, 27.5 keV), which have sufficiently distant spectral peak energies to avoid substantial interference. Even in this case, if the dose to the target object is low, contamination from other tracers and Compton scattering can have a significant impact. Thus, a high signal-to-noise ratio owing to high energy resolution is beneficial. Our previous study illustrated that the energy resolutions of the NaI(Tl) (NanoSPECT^14^), CZT semiconductor detector (X-SPECT^15–17^) and CdTe semiconductor detector (CdTe-DSD SPECT-I^8^) are 22.7%, 22.5% and 7.5% at 20–28 keV, and 8.7%, 5.3% and 1.6% at 140 keV respectively. While CZT exhibits a good energy resolution at 140keV, but both CZT and NaI(Tl) has low energy resolution at 20-28 keV. Although the U-SPECT II^7^ with NaI(Tl) scintillator detectors has an excellent spatial resolution comparable to CdTe-DSD SPECT, its energy resolution is less favourable, especially for low-energy X-rays. In contrast, for pairs restricted to high-energy radionuclides only, scintillators can provide good results by referring to a detailed report on multi-isotope capabilities by Lukas et al.^14^ The NanoSPECT, used by Lukas et al., does not resolve the 700 µm hole in the resolution phantom; however, a similar system, U-SPECT-II, has a higher spatial resolution as mentioned earlier. Our system has a slightly weaker spatial resolution at high energy X-rays (350–500 µm at 165–175 keV) owing to the collimator structure, along with reduced efficiency in detecting high-energy X/γ-rays. Practically, the system remains effective for up to 171 keV γ-rays of ^111^In. Therefore, U-SPECT-II is more suitable for high-energy radionuclide pairs. The choice of radioactive iodine (i.e. ^123^I and ^125^I) must also be discussed. ^123^I is essential for the development of clinical diagnostic radiopharmaceuticals. Alternatively, the complexity of handling halogens when performing SPECT should be considered. Labelling with halogens requires organic synthesis, which is to some extent more complicated than handling metal radionuclides. Moreover, ^125^I, which has a much longer half-life than ^123^I, is easier to handle, particularly for multi-radionuclide imaging where multiple tracers have to be prepared. ^125^I also provides long-term tracer observations. Depending on the situation, an improved combination of iodine (^123^I or ^125^I) and imaging system should be selected.

When radionuclides have emission lines with proximate energy levels, such as 140 keV for ^99m^Tc and 159 keV for ^123^I, methods have been reported to optimise the energy windows to minimise crosstalk and ensure adequate sensitivity^14,18,19^. Conversely, we developed a spectroscopic approach that negates the need for window optimisation using spectra obtained with a CdTe detector, enabling the identification of various radiation components, including emissions from radionuclides, Compton scattering, and fluorescence X-rays. Using spectra derived from the target radionuclide separated by this method, we do not have to consider noise spectra (photons), such as crosstalk and scattered lines, which increases the sensitivity because a wide energy window can be set; moreover, the signal-to-noise ratio is high because crosstalk and scattered lines are not included. Consequently, we can obtain quantitatively accurate images for each tracer even when the spectral peak energies are close^8,20^. This technique is somewhat cumbersome, but can be used if necessary.

High-spatial-resolution images with multiple tracers are pivotal because they reveal detailed local distributions of individual molecules, thereby facilitating the exploration of their functions and interactions. For instance, we could simultaneously observe the fine structure of the lymph tract and the micro-metastasis in the lymph node. The SPECT images correlate well with low-magnification immunofluorescence microscopy images. In breast cancer, sentinel lymph nodes are detected by various methods or modalities, such as using dyes such as blue dye or indocyanine green, ultrasound, MRI, and SPECT, and sentinel node biopsy is performed to evaluate lymph node micro-metastasis^9,10,21^. The present study indicates that high-resolution dual-tracer SPECT hold the promise for simultaneous detection of sentinel lymph nodes and lymph node micro-metastases in humans in a single examination. However, further evolution of SPECT, along with the development of diagnostic reagents is imperative for practical applications.

High-spatial-resolution multi-radionuclide *in vivo* imaging has numerous potential applications. Given that a typical animal cell measures 10–20 µm in diameter, the method explored in this study can be employed to analyse microstructures and morphology, consisting of a few dozen cells, concerning the distribution of multiple molecules. This is especially advantageous in smaller animals such as mice. The importance of diagnostic imaging using radiotracers has recently increased. For example, several diagnostic tracers have been developed for dementia^22,23^. In addition, the emergence of radiotheranostics, targeted radionuclide therapy based on molecular imaging techniques^24^, has stimulated the development of radioactive tracers. The demand for multi-radionuclide imaging may increase with the number of useful radioactive tracers. We expect that this method will contribute to biomedical research involving small animal imaging, such as drug development^25^ and disease research^26^ as a modality of choice for molecular imaging.

## Methods

### General set-up of SPECT

The CdTe-DSD SPECT-1 employed in this study was reported in our previous work^8^. CdTe-DSD SPECT-1 has eight CdTe semiconductor detectors, each 32 mm wide and 0.75 mm thick, placed axisymmetrically to the origin of the coordinates, and a multi-pinhole collimator composed of tungsten alloy (W-Cu-Ni) with a density of 17.8 g/cm^3^. We placed 21 pinholes of 200 μm diameter per detector. The imaging band of CdTe-DSD SPECT-I was targeted to cover low-energy X-rays (23.2 keV and high-energy gamma rays (171 keV) from ^111^In. The field of view (FOV) of CdTe-DSD SPECT-1 was approximately 12 mm in diameter. Images of the Derenzo phantoms with holes having a minimum diameter of 350 µm showed that the system could resolve the 350 µm holes. For animal imaging, CT scans were initially performed using a NanoSPECT/CT scanner (NanoSPECT 4R/CTT, Mediso, Budapest, Hungary) before SPECT imaging with the CdTe-DSD SPECT-1. The energy window of ^125^I for a single radionuclide was set at 20–38 keV. For dual-radionuclide imaging, the windows were set to 25–30 keV for ^125^I and 138–142 keV for ^99m^Tc. Maximum likelihood expectation maximisation (MLEM) with 10 iterations was used for the image reconstruction. The number of voxels defining the object space was 118 × 198 × 198 = 4626072 and the size of each voxel was 0.125 × 0.125 × 0.125 mm^3^.

### Reagents

The medium and reagents were purchased from FUJIFILM Wako Chemicals (Osaka, Japan) and Merck (Darmstadt, Germany), respectively, unless otherwise mentioned. ^99^Mo-^99m^Tc-generator was purchased from FUJIFILM Toyama Chemical Co., Ltd. (Ultra-Techne-Kow, Tokyo, Japan) and ^125^I-NaI was purchased from PerkinElmer Inc. (NEZ033A, Iodine-125 Radionuclide, MA, USA).

### Cell culture

A murine NIS-overexpressing stable clone of the mammary carcinoma 4T1 cell line (4T1-mNIS) was purchased from Imanis Life Sciences (MN, USA). 4T1-mNIS cells were cultured in RPMI 1640 medium supplemented with 10% foetal bovine serum under an atmosphere of 5% CO_2_ in air at 37 °C.

### Spheroid imaging

4T1-mNIS cells were seeded in ultra-low attachment culture dishes at a density of 30,000 cells/mL in 10 mL of medium composed of DMEM/F12 medium containing 20 ng/mL epidermal growth factor (PeproTech EC, NJ, USA), 20 ng/mL basic fibroblast growth factor (Pepro), 200 ng/mL heparan sulphate (Merck), and B27 Supplement Minus Vitamin A (Thermo Fisher Scientific, MA, USA). The cells were cultured until spheroids were formed. On the day of SPECT imaging, approximately 20 spheroids were picked under a stereomicroscope and transferred to a microplate loaded with 100 mL stem cell medium containing 500–1000 kBq ^125^I. The spheroids were incubated for 3 h and washed thrice with HBSS. A single spheroid was picked, transferred to a microtube with a small amount of HBSS, and imaged using CdTe-DSD SPECT-1 for 10–60 min. Subsequently, activity was measured using a γ-counter. The imaging time was extended when a low activity level was expected, based on factors such as the size of the spheroid, to exceed at least 2.4 × 10^4^ counts, ensuring a statistical error of less than 2%, with a maximum duration of 60 min, as the actual activity was not known at the time of imaging.

### Dual-radionuclide imaging of phantom model of a lymph node with micro-metastasis

A microtube loaded with an aqueous solution of ^99m^Tc-pertechnetate was used as the lymph node phantom because an aqueous solution of ^99m^Tc-phytic acid has been used as a tracer for sentinel lymph nodes in clinical practice in Japan. Silver-exchanged zeolite spheres approximately 900 µm in diameter (donated by Union Showa K.K., Tokyo, Japan) were used as phantoms for the micro-metastases. The zeolite spheres were immersed in a ^125^I-NaI aqueous solution and agitated for 3 min at 20 °C. The iodine-loaded zeolite spheres were washed three times with saline. The activities of the washed zeolite sphere and the aqueous solution of ^99m^Tc-pertechnetate were individually measured using a dose calibrator, and the zeolite sphere was immersed in the aqueous solution in a microtube. The SPECT acquisition time was 60 min. The ROI was demarcated with reference to the SPECT images, and the activity was obtained from the photon counts of ^125^I and ^99m^Tc inside the ROI.

### Animal model of lymph node micro-metastasis

Young adult (6 weeks old) female mice (BALB/cAJcl nu/nu, n = 5) were obtained from CLEA Japan (Tokyo, Japan) and housed for one week prior to tumour initiation. 4T1-mNIS cells were trypsinised and resuspended, and aliquots of 1 ×10^6^ cells in 20 µL of a mixed solution of HBSS and Matrigel (1:1) were injected subcutaneously into the right foot pad. Imaging experiments were performed 4 weeks after tumour cell transplantation.

The mice were maintained in cages under specific pathogen-free conditions, provided with standard food and free access to sterilised water. All experiments were carried out in accordance with the ARRIVE guidelines (https://arriveguidelines.org). All procedures for animal experiments in this study were approved by the Institutional Review Board of the National Cancer Center (Approval No. K18-018-M2) and performed in accordance with the Guidelines for the Care and Use of Laboratory Animals.

### Dual-radionuclide imaging of micro-metastasis and the lymph tract of a mouse

A solution of ^125^I-NaI was used as a tracer for NIS-expressing tumours, whereas ^99m^Tc-phytic acid was used as a tracer for sentinel lymph nodes. During tracer injection and imaging, the mice were anaesthetised with isoflurane (induction, 3%; maintenance, 1.5%). The solution of ^125^I-NaI was administered through the tail vein 3 h before imaging, while ^99m^Tc-phytic acid was injected topically around the tumour site immediately before imaging. In the initial cohort, one of five mice exhibited potential micro-metastases on SPECT. Further experiments and analyses were conducted on this mouse. Computed tomography (CT) was used to obtain images of the lower body of the mouse. This was followed by the acquisition of image data around the right popliteal fossa using the CdTe-DSD SPECT-1. Each FOV lasted 30 min, covering three FOVs for a total duration of 90 min.

### Tissue sections and immunostaining

The right popliteal lymph node and its surrounding tissue were excised, embedded, and frozen in OCT compound (Sakura Finetek, Tokyo, Japan). Serial sections of 7 µm thickness were prepared during a period when ^125^I retained ample activity, and the activity of ^99m^Tc was substantially diminished due to decay. Sections were fixed in 10% formalin neutral buffer solution. H&E staining was performed for histological analysis. Immunofluorescent staining was performed to characterise tissue- and tumour-specific markers, and monoclonal anti-mNIS antibody (Imanis) was used for mNIS-expressing tumour cells, the anti-CD169-antibody (BioLegend, CA, USA) for macrophages, and the anti-CD45R-antibody (BioLegend) for B cells. A Keyence BZ-9000 microscope with the BZ-II-Viewer software was used for all microscopic imaging, and the images were merged using the BZ-II-Analyser software (Keyence, Osaka, Japan). Autoradiography of the sections was performed using FLA-7000 (FUJIFILM, Tokyo, Japan).

### Supplementary Information

Supplementary Information.

## Data Availability

The author declare no competing interests. All data generated or analyzed during this study are included in this published article and the Supplementary Information file.
