# Supplementary material for: Dual-radionuclide in vivo imaging of micro-metastasis and lymph tract with submillimetre resolution
Source: Sci Rep. 2023 Nov 9;13:19464. doi: 10.1038/s41598-023-46907-1 (PMC10636167; doi:10.1038/s41598-023-46907-1)

Supplementary Information

Dual-radionuclide *in vivo* imaging of micro-metastasis and lymph tract with submillimetre resolution

Atsushi Yagishita[1,*], **Shin'ichiro Takeda**[1], **Kazunobu Ohnuki**[2], **Miho Katsuragawa**[1], **Oltea Sampetrean**[3,4], **Hirofumi Fujii**[2], **and Tadayuki Takahashi**[1,5]

[1]Kavli Institute for the Physics and Mathematics of the Universe (Kavli IPMU, WPI), The University of Tokyo, 5-1-5 Kashiwanoha, Kashiwa, Chiba 277-8583, Japan
[2] Exploratory Oncology Research and Clinical Trial Center, National Cancer Center, 6-5-1 Kashiwanoha, Kashiwa 277-8577, Japan
[3] Department of Molecular Biology, Keio University School of Medicine, 35 Shinanomachi, Shinjuku Tokyo 160-8582, Japan
[4] Human Biology-Microbiome-Quantum Research Center (WPI-Bio2Q), Keio University, 2-15-45 Mita, Minato,Tokyo 108-8345, Japan
[5] Department of Physics, The University of Tokyo, 7-3-1 Hongo, Bunkyo, Tokyo 113-0033, Japan
[*]yagisitta@g.ecc.u-tokyo.ac.jp

Contents

Page

Supplementary Table 1.---3

Supplementary Table 2.---5

Supplementary Figure 1.--6

Supplementary Figure 2.--7

Supplementary Method. ---8

Supplementary Table 1. Detailed zeolite imaging data
Scale bar: 1.0 mm.

Zeolite No.	Size (μm)	Dose calibrator (kBq)	CdTe-DSD SPECT-I (CPS)	Microscopic image	SPECT image
1	788	886.4	281.36	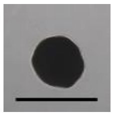	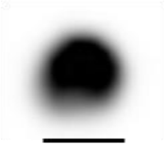
2	899	234.4	72.56	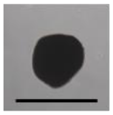	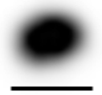
3	900	718.4	229.26	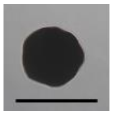	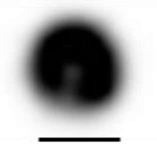
4	977	646.4	201.8	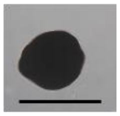	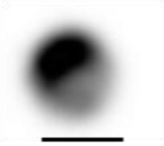
5	874	99.4	31.39	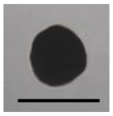	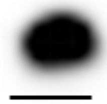
6	836	307.4	101.59	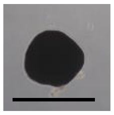	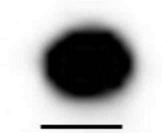
7	880	76.7	24.23	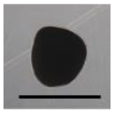	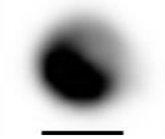
8	874	41.6	12.86	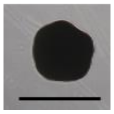	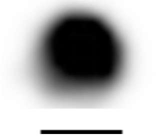

9	961	58.8	18.48	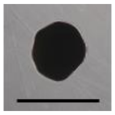	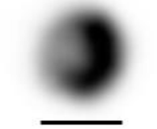
10	874	19.02	6.03	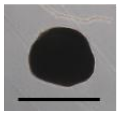	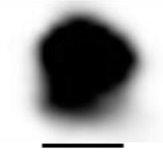
11	849	9.9	3.1	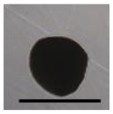	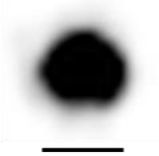
12	877	2.57	0.65	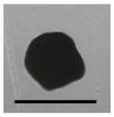	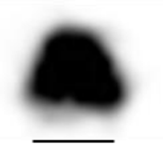

Supplementary Table 2. Detailed spheroid imaging data
Scale bars (both red and black): 500 μm; ND, not detected; NA, not available.

Spheroid	Size	(μm)	γ-counter	CdTe-DSD SPECT-I	Image	
No.	Long axis	Short axis	(kBq)	(kBq)	Microscopy	SPECT
**1**	374	181	33.48	30.22	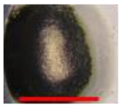	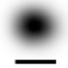
**2**	219	214	2.93	2.61	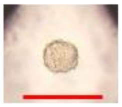	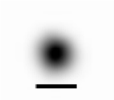
**3**	207	191	1.94	1.22	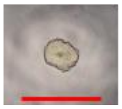	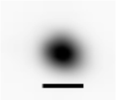
**4**	374	329	3.7	3.13	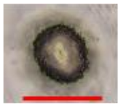	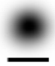
**5**	199	145	1.71	1.46	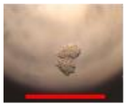	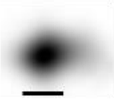
**6**	210	181	0.77	0.43	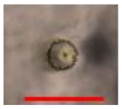	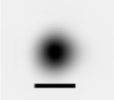
**7**	374	354	44.68	39.51	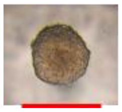	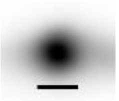
**8**	70	70	0.01	N.D.*	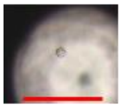	N.A.*

Supplementary Figure 1. Simulation of images with varying tumour activity. We verify the appearance of the image at lower activity of this tumour by simulation using the data from this animal experiment and the zeolite sphere data. First, we created simulated detector images by combining the detector images obtained using these two data points. We prepared three types of simulated detector images by weighting the data with the small source to be equivalent to 1 kBq, 100 Bq, and 50 Bq. Then, we reconstructed these simulated detector images. At 100 Bq, the difference from the surroundings is discernible, whereas at 50 Bq, the image was not discernible from the background.

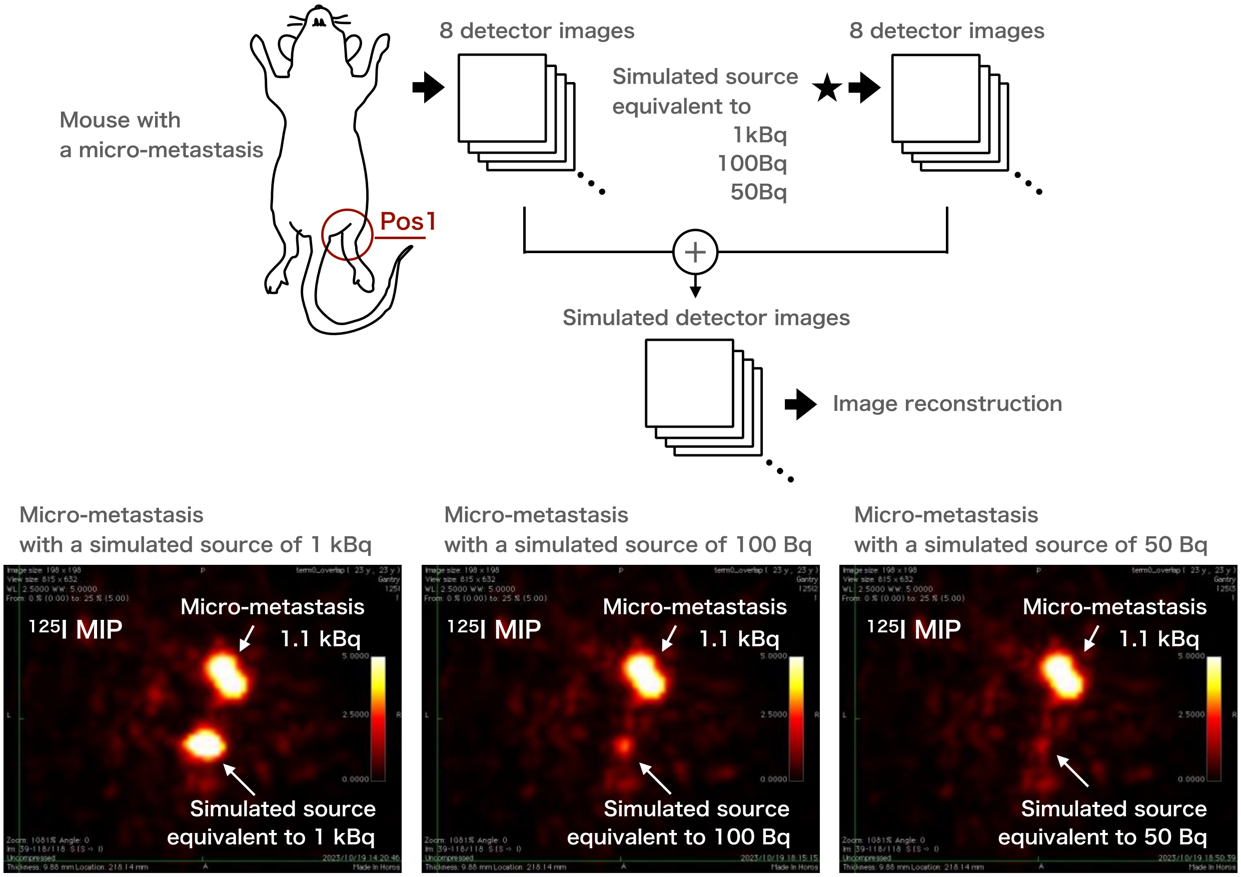

Supplementary Figure 2. Dual-radionuclide *in vivo* imaging of a cancer-bearing mouse with a lymph node metastasis. **a** SPECT(/CT) image slices of the right leg of the cancer-bearing mouse in the coronal view. Scale bar: 2.0 mm. **b** SPECT maximum intensity projection (MIP) image corresponding to the image in **a**. Scale bar: 2.0 mm. **c** Haematoxylin and eosin (H&E) staining of the popliteal lymph node (LN) and the surrounding tissues. The dashed square area corresponds to the immunofluorescence image area of **d**. Scale bar: 2.0 mm. **d** Immunofluorescence staining of the serial section. The NIS-positive metastatic tumour (yellow) is surrounded by immune cells (B220/CD45R-positive (red) B cells and CD169-positive (green) macrophages) in the LN. Scale bar: 2.0 mm. **e** Autoradiograph of a serial section, showing a high-intensity spot at the tumour site. **f** Spectrum obtained with CdTe-DSD SPECT-I.

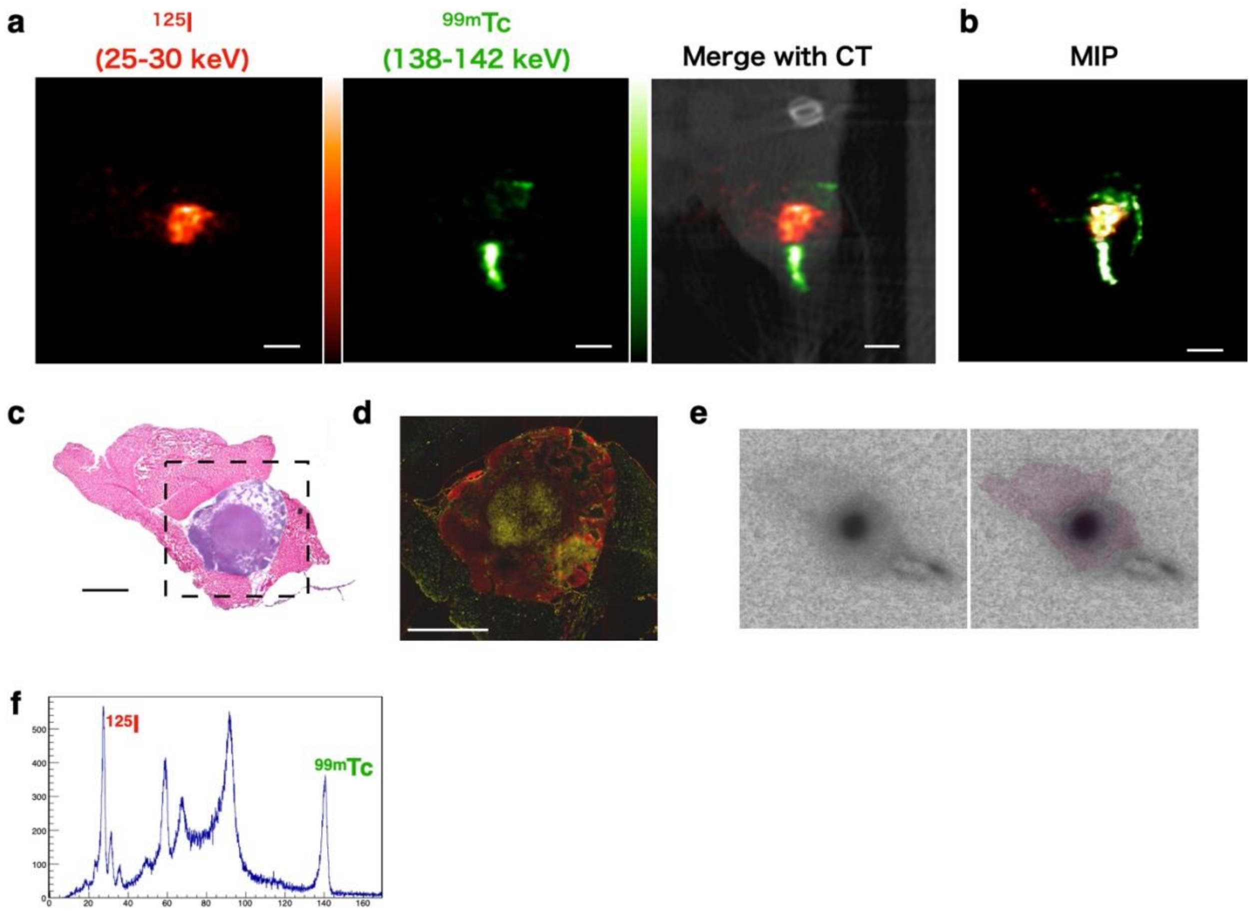

Supplementary method. Evaluation of uncertainties of the activity of the metastatic lesion.

The uncertainty in the measured activity of the metastatic lesion originates from photon statistics, error propagation during image reconstruction, and photon attenuation in the body. First, we evaluated the uncertainty due to the former two factors, photon statistics and image reconstruction, by simulation. Thirty statistically independent datasets were created by dividing the data obtained from a source with a similar size to the metastatic lesion into the same time interval. The time interval was determined so that each data point had photon statistics corresponding to those of the metastatic lesion. We generated 30 reconstructed images from the datasets and evaluated the variance of source activities in the images. The variance was approximately 5%, indicating that the uncertainty in photon statistics and image reconstruction was approximately 5%.

We then estimated the uncertainty due to photon attenuation in the body by evaluating the attenuation using a CT image that overlapped with our SPECT image. From the axial CT slice of the right leg where the micro-metastasis existed, the diameter of the right leg was approximately 10 mm, which means that the length of the object shielding the X-rays was a maximum of 5 mm in the deep region. In the deep region, the attenuation was estimated to be approximately 20% by assuming H_2O as the material of the object. Thus, the measured activity was underestimated by up to approximately 20%, because we did not apply an attenuation correction algorithm.

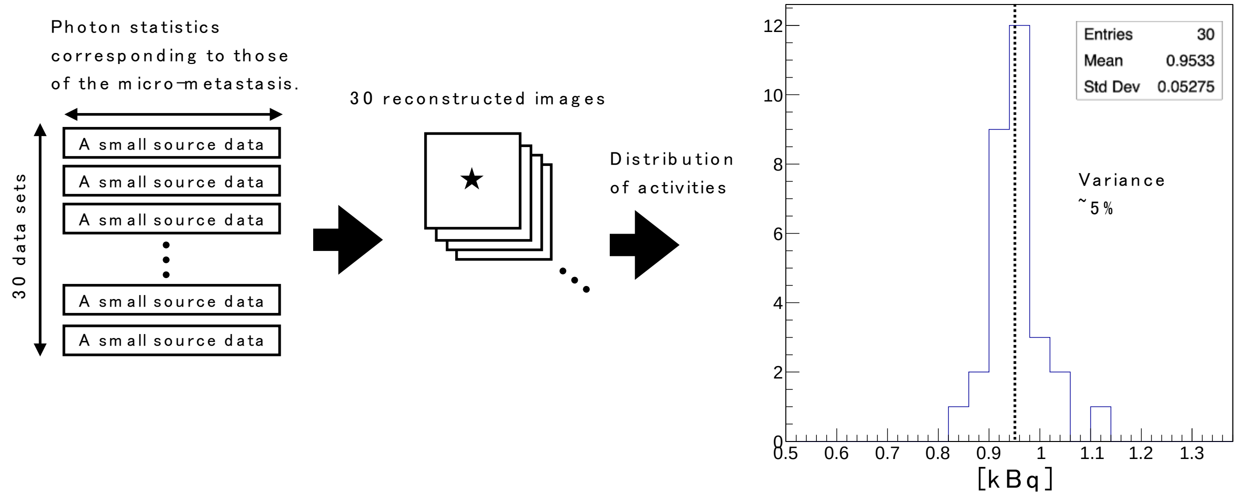

Supplement: Supplementary file 1 — Supplementary Information. [file 41598_2023_46907_MOESM1_ESM.pdf]
